# Combined Noncoding RNA-mRNA Regulomics Signature in Reprogramming and Pluripotency in iPSCs

**DOI:** 10.3390/cells11233833

**Published:** 2022-11-29

**Authors:** Salam Salloum-Asfar, Sara A. Abdulla, Rowaida Z. Taha, I. Richard Thompson, Mohamed M. Emara

**Affiliations:** 1 Neurological Disorders Research Center, Qatar Biomedical Research Institute, Hamad Bin Khalifa University, Qatar Foundation, Doha P.O. Box 34110, Qatar; 2 Qatar Biomedical Research Institute, Hamad Bin Khalifa University, Qatar Foundation, Doha P.O. Box 34110, Qatar; 3 Basic Medical Sciences Department, College of Medicine, QU Health, Qatar University, Doha P.O. Box 2713, Qatar

**Keywords:** noncoding RNAs, miRNAs, piRNAs, snoRNAs, iPSCs, reprogramming, pluripotency, differentiation

## Abstract

Somatic cells are reprogrammed with reprogramming factors to generate induced pluripotent stem cells (iPSCs), offering a promising future for disease modeling and treatment by overcoming the limitations of embryonic stem cells. However, this process remains inefficient since only a small percentage of transfected cells can undergo full reprogramming. Introducing miRNAs, such as miR-294 and miR302/3667, with reprogramming factors, has shown to increase iPSC colony formation. Previously, we identified five transcription factors, GBX2, NANOGP8, SP8, PEG3, and ZIC1, which may boost iPSC generation. In this study, we performed quantitative miRNAome and small RNA-seq sequencing and applied our previously identified transcriptome to identify the potential miRNA–mRNA regulomics and regulatory network of other ncRNAs. From each fibroblast (N = 4), three iPSC clones were examined (N = 12). iPSCs and original fibroblasts expressed miRNA clusters differently and miRNA clusters were compared to mRNA hits. Moreover, miRNA, piRNA, and snoRNAs expression profiles in iPSCs and original fibroblasts were assessed to identify the potential role of ncRNAs in enhancing iPSC generation, pluripotency, and differentiation. Decreased levels of let-7a-5p showed an increase of SP8 as described previously. Remarkably, the targets of identifier miRNAs were grouped into pluripotency canonical pathways, on stemness, cellular development, growth and proliferation, cellular assembly, and organization of iPSCs.

## 1. Introduction

The generation of induced pluripotent stem cells (iPSCs) is dependent on the reprogramming of somatic cells by forced introduction of the reprogramming factors into the cell [1]. However, the efficiency of this induced reprogramming is still low, and different factors have been reported to be implicated in determining the efficacy of this process [2,3]. One of these factors is non-coding RNAs (ncRNAs), described as RNA molecules that are not translated into proteins but are widely recognized as housekeeping molecules and master regulators of gene expression transcriptionally and post-transcriptionally [4,5]. NcRNAs can either be classified according to the transcript size or their function into two types; (i) small ncRNAs (sncRNAs; <200 nucleotides) and long ncRNAs (lncRNAs; >200 nucleotides) or (ii) housekeeping/structural ncRNAs and regulatory ncRNAs [6].

Among the sncRNAs, miRNAs are short non-coding transcripts of ~22 nucleotides that act as negative regulators for post-transcriptional gene expression. This action can be done by forming an RNA-induced silencing complex that binds to the 3′ untranslated region (UTR) of the target messenger RNAs (mRNAs), which further causes either mRNA degradation or translational inhibition [7]. Another type of sncRNAs is the piwi-interacting RNAs (piRNAs), a class of animal-specific small silencing RNAs of ~21–35 nucleotides [8,9], which can function only if they are associated with PIWI proteins. This association forms the piRNA-induced silencing complexes, hence, silencing their targets at transcriptional and post-transcriptional levels [5]. In addition, most animals have a defending mechanism through a subset of piRNAs against transposon mobilization to preserve the germline genome [5,8]. A further type of small ncRNAs is called small nucleolar RNAs (snoRNAs) which are transcripts of ~60–300 nucleotides in length and predominantly accumulated in the nucleolus [10,11]. There are two classes of snoRNAs: C/D box snoRNAs that guide-2′-O-ribose methylation and H/ACA box snoRNAs that direct the pseudouridylation of nucleotides [10,11]. A well-known function of these ncRNAs is the processing of ribosomal RNA (rRNA), including rRNA modification, maturation, and stabilization. Additionally, snoRNAs have been implicated in regulating gene expression through mRNA splicing and editing [11].

Most of the above-mentioned cellular processes that are regulated by ncRNAs are known to be involved in mediating stem cell pluripotency. Therefore, several studies have focused on identifying miRNAs that may have a role in maintaining pluripotency and enhancing the generation of iPSCs through the different stages of induced reprogramming. These miRNAs were classified according to their mode of action within a cell during the reprogramming process [12]. A group of miRNAs (miR-21, miR-29a, let-7a, and miR-34) was identified at the beginning of the reprogramming process and act as a protector to reserve the genomic integrity and somatic identity of the original cell [13]. Other miRNAs (miR-155, miR-10b, miR-205, and miR-429) were found to modulate the epithelial-mesenchymal/mesenchymal-epithelial transition (EMT/MET), which is essential during the reprogramming process and for the transformation into a pluripotent state [14]. Moreover, miRNAs that belong to the miR-290/302 seed family are found within the pluripotency regulatory network that maintains the pluripotent state of iPSCs [12,15]. It has been shown that the inhibition of those miRNAs reduces the reprogramming process, whereas the introduction of enhancer miRNAs improves the efficiency of the iPSC generation [16]. In line with this, those miRNAs that are involved in the reprogramming process and maintaining pluripotency are mainly activated via one of the crucial reprogramming factors, c-Myc. c-Myc activates the ESC-specific cell-cycle regulating (ESCC) miRNAs (e.g., miR-294) that contribute to the unique cell cycle of ESCs and were found to enhance reprogramming efficacy [17].

However, piRNA and snoRNAs are less known to have a role in reprogramming and pluripotency. One study has identified three piRNA families expressed in reprogrammed stem cells, and its analysis suggested that these piRNAs are implicated in the reprogramming process but not cellular pluripotency. Moreover, piR-64162 has been reported to regulate cell senescence during the reprogramming process [9]. The role of snoRNAs in reprogramming and pluripotency is proposed to be indirect through ESC snoRNAs that guide the dyskerin ribonucleoprotein complex (DKC1 identified as OCT4/SOX2 coactivator) to the gene enhancer. Thus, both snoRNAs and DKC1 are believed to play a role in the transcriptional regulatory network of core pluripotency genes [18,19].

In our previous work, we generated 12 clones of iPSCs from four different primary fibroblasts and found that one of these samples, A53T-PD2, showed significantly higher reprogramming efficiency. To investigate the potential cause behind the elevated reprogramming efficiency, we analyzed the RNA-seq data of each sample and identified five potential transcription factors that may boost iPSC generation [20]. In this study, we performed miRNAome and small RNA-seq sequencing and applied our previously generated transcriptome to identify the potential miRNA–mRNA regulomics and regulatory network of other ncRNAs. Additionally, we compared the miRNA, piRNA, and snoRNAs expression profiles in the generated iPSCs and the original fibroblasts to identify the potential role of ncRNAs in enhancing iPSC generation, pluripotency, and differentiation.

## 2. Materials and Methods

### 2.1. RNA Samples 

**Figure 1:**
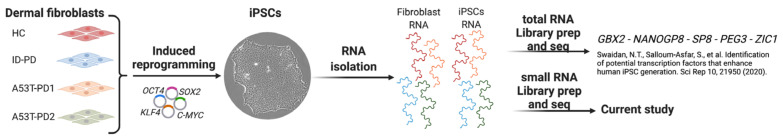
Previous research workflow and results [20]. Using Sendai Virus methodology, we reprogrammed four fibroblast lines from participants from whom appropriate written consent was obtained. Reprogrammed iPSC lines formed compacted colonies and exhibited a high nucleus to cytoplasm ratio. Based on immunocytochemistry (ICC) and differential gene expression analysis, all reprogrammed iPSC lines expressed pluripotency markers and were capable of differentiating into cells of all three germ layers. RNA sequences were conducted on the four fibroblasts and their well-characterized and validated iPSC lines.

The dermal Fibroblasts, HC, ID-PD, A53T-PD1, and A53T-PD2 were used in this study and were obtained from our previous study (Figure 1) [20]. The methodology used for reprogramming the fibroblasts into iPSCs, characterization, validation, and RNA sequencing of the fibroblasts and their reprogrammed iPSCs is described in our previous publication [20].

### 2.2. Small RNA Library Preparation and Sequencing

Using 500 ng of total RNA, small RNA libraries were constructed. In total, 52 Qiagen miRNA library QC spike-ins (Qiagen, cat. No. 331541) were used as an internal control for miRNA expression. The libraries and molecular indexes were constructed using QIAseq miRNA NGS 96 Index IL (Qiagen, Cat. No. 331565) and QIAseq miRNA NGS 96 Library Kit (96) (Qiagen, Cat. No. 331505). cDNA was created by reverse transcription of small RNA with 3′ and 5′ adaptor ligations targeting miRNAs. In reverse transcription, both 3′ and 5′ adaptors are added to the RNA fragments. A UMI (Unique Molecular Indices) was incorporated into the reverse transcription primer. RT primers bind to a region of the 3′ adapter and facilitate the conversion of 3′/5′ miRNAs into cDNA while assigning a UMI to each miRNA. A universal sequence is also added during reverse transcription. Sample indexing primers recognize this sequence during library amplification. To purify cDNA constructs, magnetic beads were used. Afterwards, binomial amplification of libraries was achieved using dried universal forward primers paired with 96 dried reverse primers (Qiagen, Cat. No. 331565). Consequently, each sample was assigned a unique custom index. Following the amplification of the library, a cleanup was conducted using the streamlined magnetic bead-based method. An Agilent technology 2100 Bioanalyzer (Doha, Qatar) and Agilent High Sensitivity DNA assay (Cat. No. G2938-90020) were used to validate the libraries. Only one peak of approximately 141 base pairs was observed.

The average size of the cDNA libra”Ies’was determined by the bioanalyzer and the Qubit Fluorometer, Qubit HS dsDNA Assay Kit (Life Technologies, Cat. No. Q32854). The libraries were diluted to 10 nM using a resuspension buffer and pooled using unique indexing for Illumina. A final dilution of 3 nM was loaded, followed by clustering on cBot2 and sequencing by Illumina using the HiSeq 3000/4000 SBS Kit (150 cycles). For the discovery of novel miRNAs, we aimed to generate up to 20 million reads per sample.

### 2.3. Sequencing Read Mapping and Small RNA Annotation

The raw sequencing files from the Illumina HiSeq 3000/4000 in the BCL format were converted to the FASTQ format using the bcl2fastq v1.8.4 conversion program. The reads were filtered, and the adapters were trimmed. The data was evaluated for quality using FASTQC to filter out reads with a high-quality score following adapter trimming.

### 2.4. sncRNAs Analysis Using WiND Workflow

Following the principles detailed in the WiND workflow [21], the RNA sequencing data were analyzed for piRNA and other sncRNAs. RNASeq was aligned to the GRCh38 genome using STAR, followed by FeatureCounts from the Rsubread package, and, simultaneously, de Novo transcriptome counts with Salmon. According to the WiND pipeline, descriptive analyses were conducted on each count set, followed by differential expression analyses using DESeq2, limma and/or edgeR [22,23,24].

### 2.5. Differential Expression Analysis: CLC Genomics Workbench Version 20.0.4

A differential expression analysis was performed as previously described [25]. Briefly, when creating differential expression profiles, the FDR *p*-value (statistical significance) between two or more samples was calculated. The files were exported into the CLC Genomics Workbench (version 22) for reading mapping to the hg38 human genome version. As a result, a single mismatched base was reduced to 18 nucleotides. The CLC Genomics Workbench was used to analyze the results of the small RNA analysis. During mapping, filtering, and counting QIAaseq NGS spike-in reads in a dataset, a “perfect match” setting was applied. In order to normalize the NGS spike-in reads from QIAseq, they need to be multiplied by the total number of reads per sample. The Biomedical Genomics Analysis plugin of the QIAGEN miRNA Quantification workflow measured the expression of each sample miRNA found in miRBase using reads sequenced using the QIAseq miRNA Library Kit, a tool provided by QIAGEN. To assign reads to miRNAs and piRNAs, respectively, and to exclude those from further analysis, the reads were first mapped to miRBase version 22 database (http://www.mirbase.org; accessed on 1 April 2022) and piRNABank database Human_piRNA_sequence_v1.0 (http://www.regulatoryrna.org/database/piRNA/; accessed on 1 April 2022) databases. Using RNA-seq analysis, reads from the QIAseq miRNA quantification workflow were collected and mapped into non-coding RNAs, such as snoRNAs. QIAseq miRNA quantification allows for the grouping of miRNA either as mature miRNAs, in which case the same mature miRNA may be derived from different precursor miRNAs, or as seed miRNAs, in which case the same seed sequence may be detected in other mature miRNAs. The expression tables can be classified into two groups: groups based on maturity and groups based on seed. Integrated unique molecular indicators (UMI) analysis allows quantification of individual miRNA molecules, which eliminates PCR/sequencing bias. As part of the differential expression analysis, miRNAs were defined as statistically differentially expressed if they had an expression of at least 50 read counts at an absolute fold change greater than two and an adjusted *p* < 0.05. Additionally, a custom database for piRNAs was constructed.

### 2.6. miRNA Profiling Comparison and Functional Enrichment Tests

IPA (Ingenuity Pathway Analysis) was used to examine pathways and molecular networks to test candidate miRNAs’ functional enrichment. The IPA system provides a more comprehensive pathway resource. Due to the extensive information provided by IPA, it can also be used to analyze pathway crosstalk, since almost all molecules are represented, as well as their connections. IPA utilizes Fisher’s exact test to identify pathways that are enriched with miRNAs of interest. In addition, the IPA system looks for significant molecular networks in a commercial knowledge base that combines literature, gene expression, and gene annotation information. Moreover, the analyses included in Analysis Match were generated in IPA from more than 100,000 highly curated and quality-controlled human disease and oncology datasets re-processed from SRA, GEO, Array Express, TCGA (by mutational status), LINCS, GTEx, ENCODE Consortium, and more. These datasets were generated by QIAGEN’s OmicSoft acquisition, and are the “comparisons” found in DiseaseLand, OncoLand, SingleCellLand, and Normal Cells and Tissues, representing various contrasts such as disease and normal, treatment vs. non-treatment, and much more. Matches against your own analyses, analyses shared with you, and IPA’s Example Analyses are also returned in the Analysis Match.

### 2.7. Evaluation of Other sncRNAs Expressions in Fibroblasts and Their iPSCs 

The expression of sncRNAs was assessed using WIND (Workflow for PIRNAs and Beyond D) [21], a bioinformatics workflow that addresses the crucial issue of small RNA annotation, allowing for reliable identification of piRNAs and other sncRNAs that have been misclassified as piRNAs in the past.

## 3. Results

### 3.1. Small RNA Sequencing Data Preprocessing and Comparison of Fibroblasts and iPSCs

In our previous study [20], we found that A53T-PD2 fibroblasts showed the highest rate of reprogramming when compared with other fibroblasts (A53T-PD1, ID-PD, and HC). To compare and assess the data distribution of sncRNAs in the samples of newly performed small RNA sequencing data, we used WIND (Workflow for PiRNAs and Beyond D) prior to the DE analysis workflow, which consisted of count normalization, and which is necessary to make accurate comparisons of gene expressions between samples. Normalization is the process of scaling raw count values to account for the other factors. The distributions of the samples before and after data cleaning or normalization are shown in Supplementary Figure S1. In this way, the expression levels are more comparable between and within samples. The comparison between raw data and filtered data is based on the thresholding of the data based on the mean and median of the data (log2(10/median(library_size) * 1 × 10^−6^ + 2/mean(library_size) * 1 × 10^−6^) in fibroblasts and iPSCs, as shown in Supplementary Figure S1A–D, respectively. Comparing between samples, none appear to have abnormal distributions. Supplementary Figure S1 shows the effects of normalization on the filtered data for fibroblasts (Supplementary Figure S1A) and iPSCs (Supplementary Figure S1B). A multidimensional scaling (MDS) was performed to visualize the level of similarity and clustering based on gene expression. The MDS plots show that A53T-PD2 has a different distribution from other fibroblasts (Supplementary Figure S1B). Moreover, A53T-PD2 iPSCs clones are also clustered together (Supplementary Figure S1E). Furthermore, the density plots of log count distribution of normalized data showed similar and reliable distributions between the samples when compared to the raw data in fibroblasts (Supplementary Figure S1C) and iPSCs (Supplementary Figure S1F).

### 3.2. miRNA and piRNA Profiles Are Differentially Expressed in Fibroblasts vs. Their Reprogrammed iPSCs

**Figure 2:**
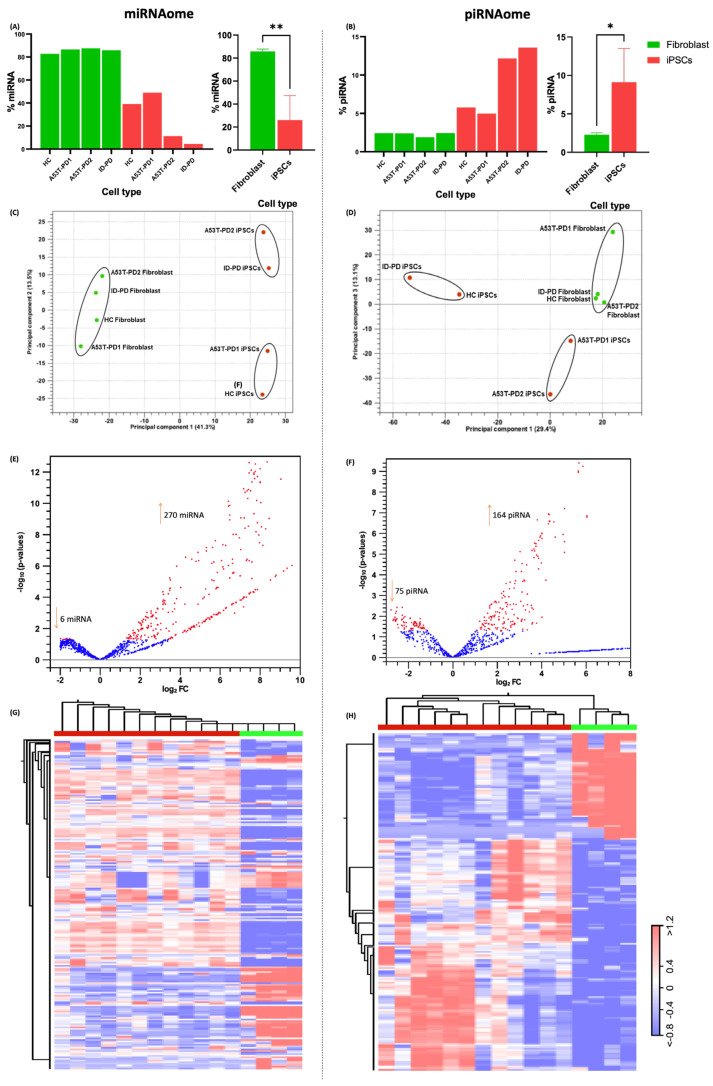
miRNA and piRNA differential expression analysis between fibroblasts and their reprogrammed iPSCs. (**A**) Bar graph showing that all fibroblasts samples express similar percentage of miRNAs, and it is significantly higher from miRNA expression in their iPSC samples (*p*-value = 0.0015; *p*-value < 0.01 corresponds to **). (**B**) piRNA expression in hiPSCs is significantly higher than their fibroblast samples (*p*-Value = 0.0208; *p*-value < 0.05 corresponds to *). (**C**,**D**) Principal component analysis (PCA) based on DE miRNAs and piRNA found between fibroblasts and their reprogrammed iPSCs. (**E**,**F**) Volcano plots of total DE miRNAs and piRNA found between fibroblasts and their reprogrammed iPSCs, respectively. (**H**) heat map of DE miRNAs in fibroblasts vs. iPSCs. (**G**) heat map of DE piRNAs in fibroblasts vs. iPSCs.

In order to identify the ncRNAs that were expressed by the samples, we first focused on those miRNAs and piRNAs that were differentially expressed between fibroblasts and the corresponding iPSCs (Figure 2A,B). Using gene expression signatures, we were able to demarcate fibroblasts from iPSCs. Based on read depth coverages, fibroblast samples exhibited significantly higher levels of miRNAs (80%) when compared to their corresponding iPSCs (20%) (Figure 2A, left and right panels). In contrast, piRNA expression in fibroblasts was only 2% (Figure 2B, left panel), which is significantly lower than the piRNA abundance in iPSCs, which is 10% (Figure 2B, right panel). In addition, we utilized two-dimensional PCA analysis to demonstrate how clearly fibroblasts and iPSCs are clustered based on their gene expression. Using miRNAs and piRNAs as indicators of expression, fibroblast samples had a distinct cluster from that of iPSCs (Figure 2C,D, respectively). Next, we performed a differential expression analysis to determine which miRNA and piRNA are expressed at different levels between fibroblasts and their reprogrammed iPSCs. Following the criteria, FDR corrected *p*-value ≤ 0.05 and FC-abs ≥ 2.0, and we identified 276 significantly DE miRNAs (270 miRNA upregulated and 6 downregulated) and 239 DE piRNAs (164 piRNA upregulated and 75 piRNA downregulated) between fibroblasts and their reprogrammed iPSCs as shown in the volcano diagram (Figure 2E,H; Tables S1 and S2). Then, we organized the DE miRNAome and piRNAome data in a heatmap to visualize the distinct expression profile between fibroblasts and their iPSCs. The hierarchical clustering demonstrated that the miRNA and piRNA profiles from fibroblasts and their iPSCs groups were distinctly separated (Figure 2G,H).

### 3.3. Fibroblasts Differentially Expressed miRNA, miRNA Target Prediction, and Interaction with Transcriptome

**Figure 3:**
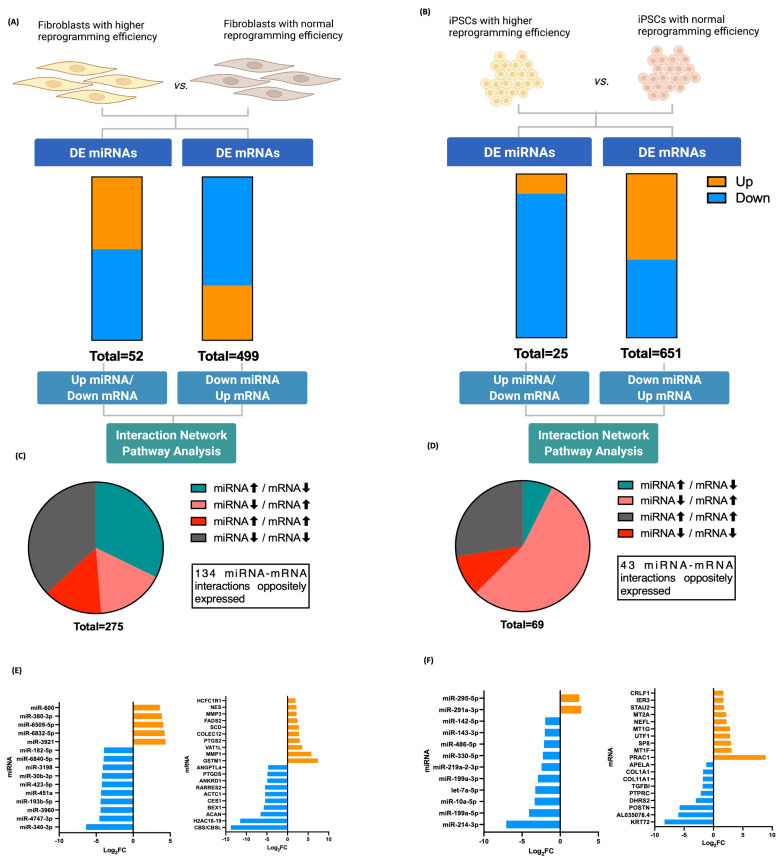
The differentially expressed miRNA and proportion estimates of DE miRNome and DE transcriptome in fibroblasts and iPSCs with high reprogramming efficiency. (**A**) A bar graph of significantly the number of down- and up-regulated miRNAs and mRNAs in fibroblasts. (**B**) A bar graph of significantly the number of down- and up-regulated miRNAs and mRNA in iPSCs. (**C**) A pie chart of miRNA-mRNA interactions in fibroblasts. (**D**) A pie chart of miRNA-mRNA interactions in iPSCs. (**E**) Top down- and up-regulated miRNAs and mRNAs in fibroblasts. (**F**) Top down- and up-regulated miRNAs and mRNAs in iPSCs.

In order to identify miRNA and mRNA interactions that occur before the iPSC reprogramming process, the DE miRNAome and DE transcriptome in fibroblasts were first identified in the A53T-PD2 fibroblasts, which have shown to have the highest rate of reprogramming, compared to the rest of fibroblasts. Only miRNAs and mRNAs with reads greater than 50 in all samples were considered for differential gene expression analysis. The t-statistics and expression fold change (FC) analysis, using *p*-values < 0.05 and absolute-FC > 2, revealed an overall differential expression of 52 DE miRNAs (23 up and 29 down) and 499 DE mRNAs (332 down- and 167 up-) in the A53T-PD2 fibroblasts (with the highest rate of reprogramming) compared to the rest of fibroblasts (Figure 3A; Tables S3 and S4). Having established the miRNAs and mRNAs that showed dysregulation in A53T-PD2 fibroblasts, we composed the mRNA targets of these DE miRNAs, using the computational miRNA target prediction IPA (Ingenuity Pathway Analysis) that uses TargetScan-Human, TarBase and miRecords databases, and experimentally validated interactions. The analysis revealed a total number of 275 miRNA–mRNA interactions, where 89 of these interactions were up-regulated (up) miRNAs and down-regulated (down) mRNAs, 45 were miRNA down/mRNA up, 39 miRNA up/mRNA up, and 102 miRNA down/mRNA down (Figure 3C). Out of all these interactions, a total of 134 miRNA–mRNA interactions were selected from direct interactions that were oppositely expressed. Fifty-five of these interactions were not predicted to be involved in pathways, whereas 79 were predicted in different pathways. The later interactions correspond to 52 microRNA, with targeting information available and filtered to 43 family miRNA clusters targeting 79 mRNAs. Out of these 43 family clusters, 4 miRNA clusters (9% of all miRNA), miR-6758-5p, miR-5191, miR-466d-5p, and miR-423-5p targeted 31 mRNAs (which corresponds to 40% of all predicted mRNAs) (Table S7). The top DE miRNAs were miR-340-3p, miR-4747-3p, miR-3960, miR-193b-5p, miR-451a, miR-423-5p, miR-30b-3p, miR-3198, miR-6840-5p, miR-182-5p, miR-3921, miR-6832-5p, miR-6509-5p, miR-380-3p, and miR-600 (Figure 3E).

**Figure 4:**
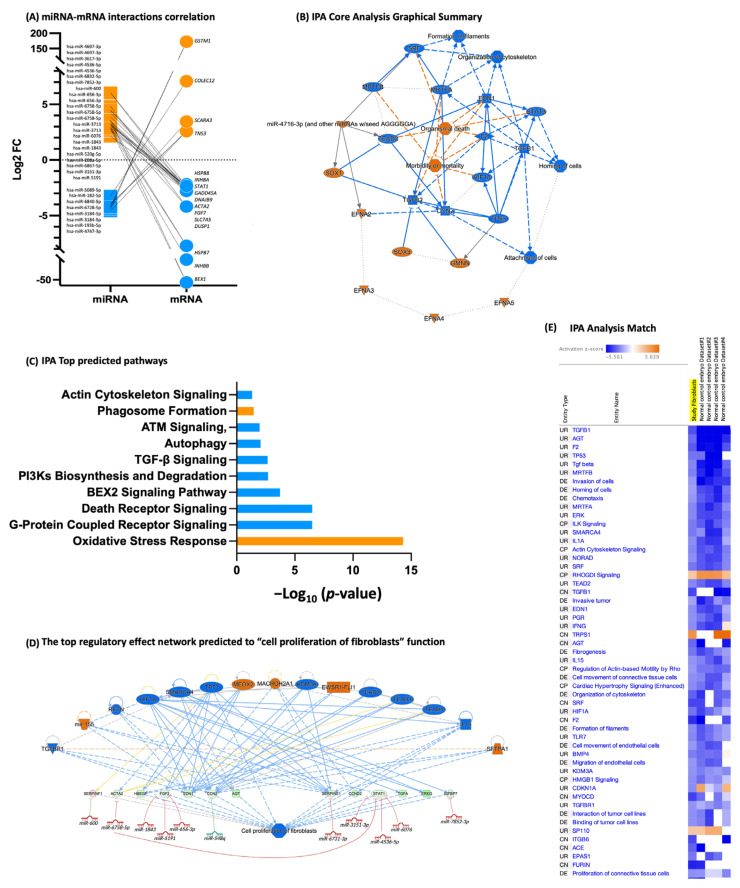
miRNA–mRNA interactions and predicted pathways in fibroblasts with high reprogramming efficiency. (**A**) miRNA-mRNA interactions correlation. (**B**) Graphical summary of the major biological themes (canonical pathways, upstream regulators, diseases, and biological functions) in the IPA Core Analysis to illustrate how those concepts relate to one another. Predicted canonical pathways encoding homing of cells, organization of the cytoskeleton, cell death, and survival are shown. The genes shaded in orange are upregulated and those that are blue are downregulated. (**C**) A bar graph of the top predicted pathways; orange bars correspond to upregulated pathways and blue to the downregulated ones. (**D**) The top regulatory effect network predicting the “cell proliferation of fibroblasts” function. The genes shaded in orange or red are upregulated and those that are blue or green are downregulated. The intensity of the shading shows to what degree each gene was up- or down-regulated. A solid line represents a direct interaction between the two gene products and a dotted line represents an indirect interaction. (**E**) IPA Analysis Match in the context of over 100,000 IPA analyses to determine similarities with the most significant canonical pathways (CP), upstream regulators (ER), diseases and biological functions (DE) and causal network (CN). The activation z-score is a summary value that predicts the activation (positive value: orange) or inhibition (negative value: blue) of a CP, UR, or CN based on the gene expression changes.

On the other hand, 10 significantly enriched pathways were predicted based on the miRNA interactions and the DE mRNAs, CBS/CBSL, H2AC18-19, ACAN, BEX1, CES1, ACTC1, RARRES2, ANKRD1, PTGDS, ANGPTL4, GSTM1, MMP1, VAT1L, PTGS2, COLEC12, SCD, FADS2, MMP3, NES, and HCFC1R1 (Figure 4A). The IPA graphical summary of the major biological themes (canonical pathways, upstream regulators, diseases, and biological functions) is shown in Figure 4C. This summary illustrates the IPA Core Analysis of how the major biological concepts relate to one another. The predicted canonical pathways encoding homing of cells, organization of the cytoskeleton, cell death, and survival are shown and enriched (Figure 4B). Two predicted pathways; “Oxidative stress response” and “Phagosome formation” are significantly enhanced (Figure 4C; shown in orange). However, eight pathways were significantly inhibited; “G-Protein receptor signaling”, “Death receptor signaling”, “BEX2 signaling pathway”, “PI3Ks biosynthesis and degradation”, “TGF-β signaling”, “Autophagy”, and “Actin cytoskeleton signaling” (Figure 4C; shown in blue). The top regulatory effect network significantly inhibited corresponds to “cell proliferation of fibroblasts” disease/function with a high consistency score (measure of how causally consistent and densely connected is the regulator effects network) of 18.112 (right-tailed Fisher’s Exact Test *p*-value of <0.05) (Figure 4D). The score is increased for consistent paths, those that connect an upstream regulator to a target and then to a disease or function where the two path segments are consistent with the published literature and the known and predicted states of the three nodes in that path.

To help confirm our interpretation of the results and to underly shared biological mechanisms, we performed an advanced analysis match of our dataset that corresponds to the highest reprogramming enhanced fibroblasts with other IPA Core Analyses (tens of thousands of other human expression analyses curated from public source; processed from SRA, GEO, Array Express, TCGA, LINCS, GTEx, and ENCODE Consortium) with similar biological results as compared to ours. This “analysis-to-analysis” matching is based on shared patterns of Canonical Pathways, Upstream Regulators, Causal Networks, and Diseases and Functions. Interestingly, we found that of the top human curated analyses, four datasets showed similar results to our data and corresponded to “normal embryo cells in differentiation media (Figure 4E).

### 3.4. iPSCs Differentially Expressed miRNA, miRNA Target Prediction, and Interaction with Transcriptome

**Figure 5:**
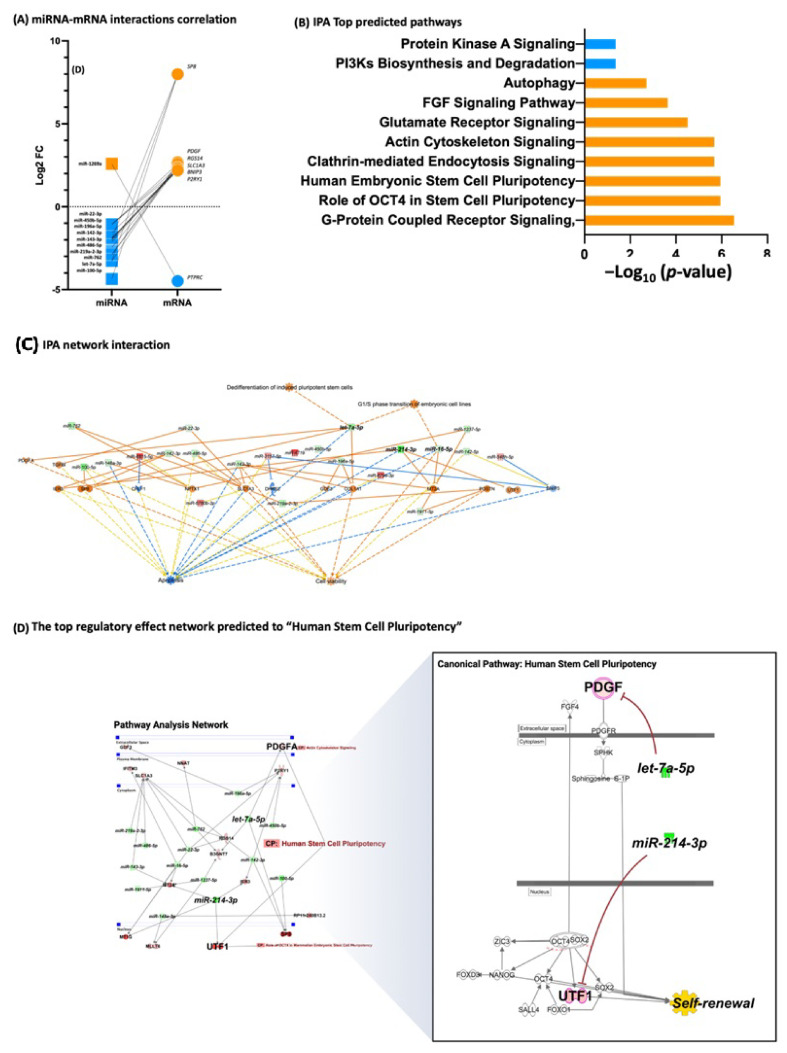
miRNA–mRNA interactions and predicted pathways in iPSCs with high reprogramming efficiency. (**A**) miRNA-mRNA interactions correlation. (**B**) A bar graph of the top predicted pathways; orange bars correspond to upregulated pathways and blue to the downregulated ones. (**C**) Predicted canonical pathways encoding stemness, cellular development, growth and proliferation, cellular assembly and organization, pluripotency, and self-renewal of human iPSCs. The genes shaded in orange or red are upregulated and those that are blue or green are downregulated. (**D**) Predicted canonical pathway encoding specifically “Human Stem Cell Pluripotency”. The genes shaded in red are upregulated and those that are green are downregulated. The intensity of the shading shows to what degree each gene was up- or down-regulated. A solid line represents a direct interaction between the two gene products and a dotted line represents an indirect interaction.

However, in the A53T-PD2 iPSCs, there were 25 miRNAs (22 down and 3 up) and 681 mRNAs (341 down and 310 up) that differed significantly from the rest of the iPSCs (Figure 3B; Tables S3 and S4). Sixty-nine miRNAs and mRNAs interactions were found of which 5 were miRNA up and mRNA down, 38 were miRNA down and mRNA up, 7 were miRNA up and mRNA down, and 19 were miRNA down and mRNA down (Figure 3D). In total, 45 interactions belonged to one pathway. As a result, we identified 43 miRNA-mRNA interactions, which corresponds to 21 families of miRNA clusters targeting 22 mRNAs (Table S8). In total, 12 mRNAs (54% of all predicted mRNAs) are targeted by the miR-142-3p, miR-214-3p, miR-22-3p, and miR-762 family clusters. The top DE mRNAs, KRT72, AL035078.4, POSTN, DHRS2, PTPRC, TGFBI, COL11A1, COL1A1, APELA, PRAC1, MT1F, SP8, UTF1, MT1G, NEFL, MT2A, STAU2, IER3, and CRLF1 are involved in stemness, cellular development, growth and proliferation, cellular assembly and organization, pluripotency, and self-renewal of human iPSCs, which were highly enriched (Figure 5). Our previous publication [20] provides a detailed description of iPSCs DE transcriptome. The top DE miRNAs were miR-214-3p, miR-199a-5p, miR-10a-5p, let-7a-5p, miR-199a-3p, miR-219a-2-3p, miR-330-5p, miR-486-5p, miR-143-3p, miR-142-5p, miR-291a-3p, and miR-295-5p (Figure 3F).

### 3.5. Differentially Expressed piRNAs and Other ncRNAs in Fibroblasts and Their iPSCs

**Figure 6:**
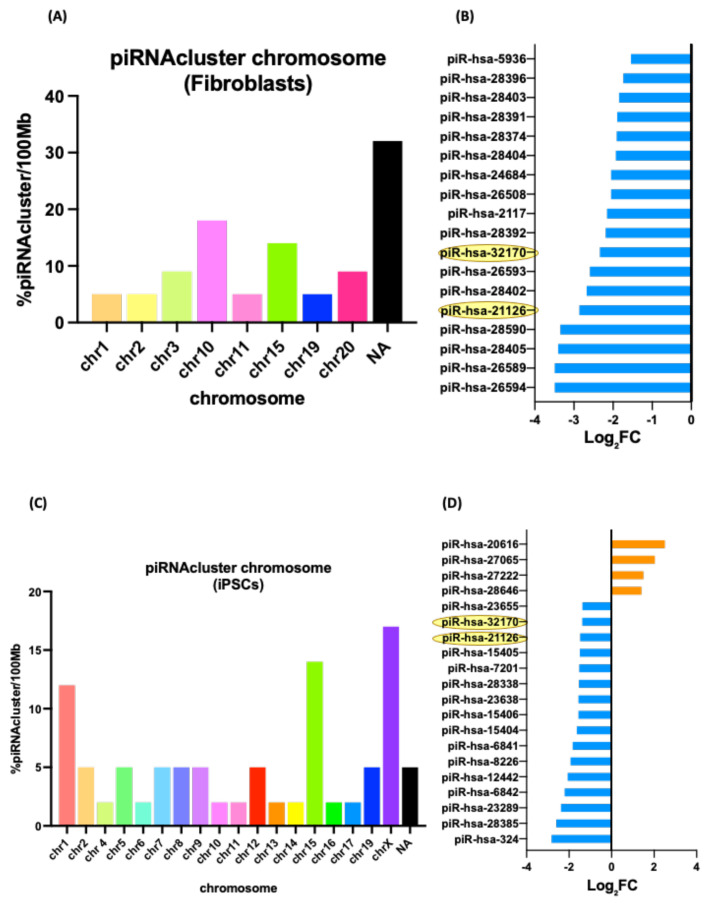
Distribution of piRNA clusters across chromosomes and differentially expressed (DE) piRNAs (**A**) piRNA cluster chromosome in fibroblasts with higher reprogramming efficiency. (**B**) DE piRNA in fibroblasts with higher reprogramming efficiency. (**C**) piRNA cluster chromosome in iPSCs with higher reprogramming efficiency. (**D**) DE piRNA in iPSCs with higher reprogramming efficiency.

Given the aim of our experimental approach to identify not only miRNAs but also other sncRNAs, we did a comparative analysis of piRNAs and other ncRNAs between fibroblasts and iPSCs with higher reprogramming efficiency. The distribution of piRNA clusters across chromosomes was variable between both groups, showing that distributions were not uniform and were not proportional to the length of the chromosome. piRNAs in fibroblasts were distributed over eight chromosomes; however, in iPSCs, they were found in 18 different chromosomes. Eighteen piRNA were upregulated in the A53T-PD2 fibroblasts, whereas 20 piRNA were DE in their iPSC counterparts. Out of those, two piRNA, piR-hsa-21126, and piR-hsa-32170 were expressed in both A53T-PD2 fibroblasts and iPSCs (highlighted in yellow; Figure 6). 

To provide functional clues, and potential target genes for the reprogramming, associated piRNAs were analyzed. Several genes neighboring piRNAs clusters were predicted: “*Protein serine/threonine kinase activity*”, *Ubiquitin-protein transferase activity*”, “*Regulation of chromatin assembly or disassembly*” in fibroblasts that showed higher efficiency in reprogramming compared to other fibroblasts, including 7 genes, GOLGA2 Pseudogene 11 (GOLGA2P11), TP53 Target 1 lncRNA (TP53TG1), Long Intergenic Non-Protein Coding RNA 661 (LINC00661), Serine/Threonine-Protein Kinase Tousled-Like 1 (TLK1), Ubiquitin Protein Ligase E3C (UBE3C), Armadillo Repeat Containing X-Linked 5 (ARMCX5), and Downstream Of Tyrosine Kinase 6 (DOK6) (Figure 6; Table 1).

In contrast, in iPSCs, several other genes neighboring piRNAs clusters were predicted: “regulation of transcription”, “multicellular organism development”, and “DNA-binding transcription factor activity”, which showed higher efficiency in reprogramming compared to other fibroblasts, including 13 genes, Zinc Finger Protein 318 (ZNF318), Protein O-Linked Mannose N-Acetylglucosaminyltransferase 2 (POMGNT2), Tight Junction Protein 1 (TJP1), Ferritin heavy chain like 18 (FTH1P18), NBPF Member 4, 6 and 7 (NBPF4, 6 and 7), Proline-Rich and Gla Domain 1 (PRRG1), Long Intergenic Non-Protein Coding RNA 205 (LOC642852), Myosin Heavy Chain 3 and 4 (MYH3 and 4), Serine/Threonine-Protein Kinase Tousled-Like 1 (TLK1), Ubiquitin Protein Ligase E3C (UBE3C), Churchill Domain Containing 1 (CHURC1), Long Intergenic Non-Protein Coding RNA 1015 (LINC01015), and Zinc Finger Protein (ZNF668) (Figure 6; Table 2).

**Figure 7:**
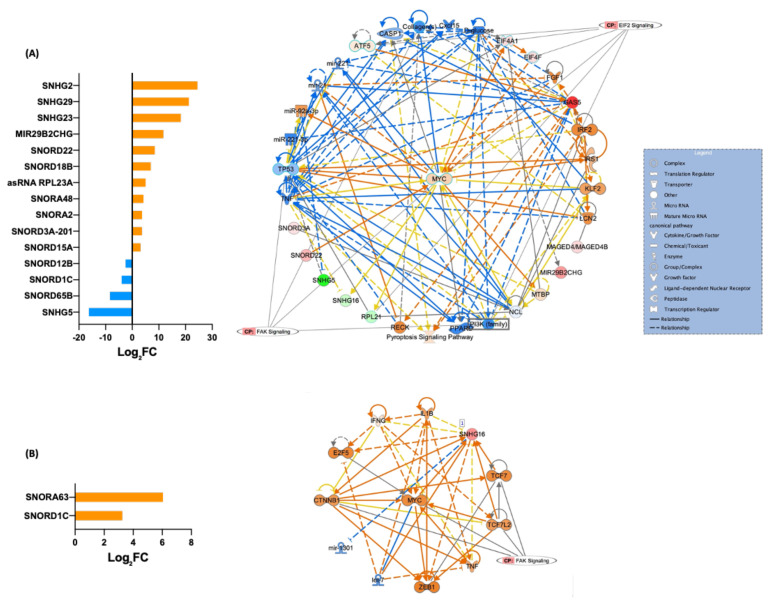
Other differentially expressed ncRNAs in fibroblasts and their iPSCs and the predicted canonical pathways. (**A**) DE snRNAs in fibroblasts with higher reprogramming efficiency and predicted canonical pathways. (**B**) DE snRNAs in iPSCs with higher reprogramming efficiency and predicted canonical pathway.

Moreover, in the Salmon’s analysis estimated for gene-level DE analysis, 15 other ncRNAs (5 lncRNA, 9 snoRNAs, and 1 snRNA) were in fibroblasts (11 upregulated and 4 downregulated). Two snoRNAs were upregulated in iPSCs; SNORA63 snoRNA, H/ACA Box 63, and SNORD1C (snoRNA, C/D Box 1C) (Figure 7). Using IPA software, two canonical pathways were predicted in fibroblasts, EIF2 signaling and FAK signaling. FAK signaling was a conserved canonical pathway in the corresponding iPSCs.

## 4. Discussion

Due to the low efficiency of iPSC reprogramming, it is often difficult to adapt the process to high throughput approaches. In this way, the identification of RNA networks at the molecular and cellular level of somatic cells is needed prior to the reprogramming process. In our previous work, we generated iPSCs from four different primary fibroblast samples and one of these samples, A53T-PD2 fibroblasts, showed a significantly higher efficiency. To investigate the potential transcription factors behind the elevated reprogramming efficiency, we performed transcriptome profiling and analyzed the RNA-seq data for each sample and three clones of iPSCs of each fibroblast. As a result, we identified five potential transcription factors (GBX2, NANOGP8, SP8, PEG3, and ZIC1) that may boost human iPSC generation [20]. In this study, we used a genome-wide sequencing approach to identify miRNA clusters, piRNAs, and snoRNAs that may function as cell modulators as fibroblasts reprogrammed into iPSCs. The role of small noncoding transcripts, miRNAs, and other sncRNAs, in diverse physiological and pathological processes is emerging, but their role in reprogramming cells is not yet fully understood. A comparison of miRNA, piRNA, and snoRNA expression profiles in iPSCs and original fibroblasts was performed to examine their role in regulating the generation of iPSCs and their pluripotency. miRNA expression profiling of iPSCs compared with their original fibroblasts provided alternative perspectives on miRNA core regulatory networks regulating differentiation and pluripotent characteristics.

It has been described that several miRNAs associated with the reprogramming of cells exhibit clustered expression, even when not encoded at the same genetic locus; that is, they are expressed simultaneously during specific normal homeostatic cellular programs [26,27]. Clusters of miRNAs are DNA regions that encode multiple miRNAs at once, illustrating the expansive nature of miRNAs as well as their functional importance. Different studies showed that the expression of multiple miRNAs is altered during reprogramming and is crucial to developing a novel and more efficient reprogramming method [28]. It is fundamental to determine whether the clustering properties of miRNAs could be exploited in this way. In this study, four miRNA clusters, miR-6758-5p, miR-5191, miR-466d-5p, and miR-423-5p, were identified to target 31 mRNAs (40% of predicted mRNAs) in fibroblasts with the highest rate of reprogramming. The functional miRNA-mRNA interactions are associated with cell homing, organization of the cytoskeleton, increased survival, and decreased cell death in fibroblasts. This fact might explain the reason behind the effectiveness and higher efficiency reprogramming effects in A53T-PD2 fibroblasts.

A53T-PD2 fibroblasts’ have correlated with increased cell homing and a decreased cell proliferation which may correlate positively to the overall reprogramming efficiency as described before [20]. Previous data in murine fibroblasts demonstrated that the proliferation rate of the somatic cell plays a critical role in reprogramming. Slowing down the proliferation and an increase of cell survival and homing properties of the original cells are described to be beneficial to the induction of the iPSCs [28,29,30].

A significant finding was that four of the top human curated analyses showed results similar to our own and corresponded to “normal embryo cells in differentiation media”. Given that we are studying the fibroblasts that showed higher rates of reprogramming, we can postulate that this phenomenon is not only specific to these fibroblast cells, but it is a general phenomenon for embryo-derived cells as well as cells with enhanced properties related to iPSC generation, pluripotency, and differentiation (Figure 4E).

The results of the miRNA identified in iPSCs A53T-PD2 clones support pluripotency properties. Remarkably, the targets of identifier miRNAs were grouped into pluripotency canonical pathways, on stemness, cellular development, growth and proliferation, cellular assembly, and organization of iPSCs. The top canonical pathway showed that A53T-PD2 iPSCs are more likely to have pro-pluripotency and self-renewal regulatory systems. In this study, we observed a decrease in miR-142-3p levels, which is previously described to regulate UTF1 [31]. This might result in an increase of iPSCs self-renewal and decreased apoptosis. miR-22-3p belongs to the Pluripotency-associated miR-290/302 family of miRNAs that promote pluripotency [32]. Moreover, miR-214-3p and miR-762, which are downregulated A53T-PD2 clones, may regulate the proliferation and differentiation [33,34].

Furthermore, the decreased levels of let-7a-5p in A53T-PD2 clones directly regulate PDGF and SP8 (Figure 5). SP8 is one of the previous transcription factors described in our previous study to enhance reprogramming. SP8 was shown to be upregulated through reprogramming and self-renewal stages. SP8 is involved in the first stages of reprogramming and is also essential in maintaining the pluripotency in later stages. Decreased levels of let-7a-5p showed an increase of SP8. Additionally, let-7a-5p decreased differentiation of induced stem cell, apoptosis, increasing cell viability. These findings are consistent with our previous research where we found that SP8 is increased in A53T-PD2 fibroblasts with higher reprogramming efficiency.

Our analyses highlighted different sncRNAs, involving piRNAs and snoRNAs, which are worth consideration. Among the analyzed piRNAs, piR-hsa-21126 and piR-hsa-32170, were conserved and downregulated in both A53T-PD2 fibroblasts and iPSCs. From an in silico analysis, there is an overlap between piR-hsa-21126 and tRNA^Ser^ gene. The tRNA^Ser^ gene is responsible for translation of a non-universal genetic code. Furthermore, piR-hsa-32170 is described as being involved in germline-specific events such as germline stem cell maintenance and meiosis [35]. Moreover, the predicted targets of piR-hsa-32170 are Serine/Threonine-Protein Kinase Tousled-Like 1 (TLK1) and Ubiquitin Protein Ligase E3C (UBE3C). TLK1 is a negative regulator of core transcription factors in murine embryonic stem cells [36]. UBE3C was described to be overexpressed in stem-like NSCLC (non-small-cell lung cancer) cells and acted as a stemness enhancer [37]. Therefore, we speculate that the downregulation of piR-hsa-32170 may effectively regulate stemness by balancing the expression of two proteins, one of which enhances stemness whereas the other inhibits it.

In germ cells, the Piwi-piRNA pathway is active, where its functions are needed for germ cell maintenance and differentiation. Piwi proteins and piRNAs have been detected outside germline tissue, but little information is available on the activity of this pathway in mammalian somatic cells. We speculated, based on our results and those of others, that piRNA might play a role in reprogramming progress via regulating genes related to senescence. Cellular senescence has been demonstrated to share some common mechanisms with cellular reprogramming and to contribute to it [9].

Although it may be difficult to speculate a possible mechanism for snoRNA/host gene CNAs contributing to disease onset, it is worth pointing out that the snoRNAs/host gene pairs shown to be altered in multiple physiological and pathological mechanisms have already been recognized for their biological relevance. The pair of snoRNA and host genes, SNORA63/EIF4A2, targets developmental potency and histone H3 transcription for translational regulation of stem cell pluripotency [38]. Indeed, by analyzing IPA data, it was possible to predict the EIF2 signaling pathway, a major component of translational regulation, as a major canonical pathway affected by the group of snoRNA and other sncRNAs (Figure 7).

It will be important to conduct further research into the regulatory and biological functions of sncRNAs and long non-coding RNAs in stem cell signaling, reprogramming, and differentiation in the future. Although a large number of ncRNAs have been identified, it has proved to be challenging to demonstrate the functional relevance of these RNAs for stem cell research, reprogramming, and differentiation. A thorough study of the ncRNA candidates involved in the pathways of pluripotency, self-renewal, and survival must be performed to determine the physiological relevance of ncRNAs in stem cell research. In order to establish a personalized approach to medicine, the ncRNA profile of each type of human stem cell should be systematically examined. Accordingly, the potential underlying mechanism of miRNA and other ncRNA-mediated reprogramming may offer an improved method of generating patient-specific iPSCs that are of improved quality and safety for regenerative medicine and transplantation therapy.

## 5. Conclusions

The significance of transcription factors involved with pluripotent stem cells for iPS cell development cannot be overstated. However, previous studies identified that miRNA clusters were able to reprogram fibroblasts more effectively than the standard OSKM (OCT4, SOX2, KLF4 and MYC transcription factors) combination reprogramming approach [26]. Considering the advancements in miRNA and ncRNAs biology, these findings may lead to a non-viral, non-transcription factor-mediated method of generating iPSCs with higher pluripotent properties. Ultimately, this could be applied for both basic stem cell biology studies and for high throughput manufacturing of human iPSCs from large populations of patients.

## Figures and Tables

**Table 1:** piRNA downregulated in fibroblasts that showed higher efficiency in reprogramming.

piRNA	Predicted Target	Predicted Target Name	Predicted Pathway
piR-hsa-26594	*GOLGA2P11*	GOLGA2 Pseudogene 11	Protein serine/threonine kinase activity/Ubiquitin-protein transferase activity/Regulation of chromatin assembly or disassembly
piR-hsa-28405	*TP53TG1*	TP53 Target 1 lncRNA	
piR-hsa-28402	*LINC00661*	Long Intergenic Non-Protein Coding RNA 661	
piR-hsa-32170	*TLK1*	Serine/Threonine-Protein Kinase Tousled-Like 1	
	*UBE3C*	Ubiquitin Protein Ligase E3C	
piR-hsa-2117	*ARMCX5*	Armadillo Repeat Containing X-Linked 5	
	*DOK6*	Downstream of Tyrosine Kinase 6	

**Table 2:** piRNA downregulated in iPSCs that showed higher efficiency in reprogramming.

piRNA	Predicted Target	Predicted Target Name	Predicted Pathway
piR-hsa-324	*ZNF318*	Zinc Finger Protein 318	Regulation of transcription/multicellular organism development/DNA-binding transcription factor activity
piR-hsa-23289	*POMGNT2*	Protein O-Linked Mannose N-Acetylglucosaminyltransferase 2	
	*TJP1*	Tight Junction Protein 1	
piR-hsa-12442	*FTH1P18*	Ferritin heavy chain like 18	
	*NBPF4, 6 & 7*	NBPF Member 4, 6 and 7	
	*PRRG1*	Proline-Rich and Gla Domain 1	
piR-hsa-8226	*LOC642852*	Long Intergenic Non-Protein Coding RNA 205	
piR-hsa-6841	*MYH3 & 4*	Myosin Heavy Chain 3 and 4	
piR-hsa-32170	*TLK1*	Serine/Threonine-Protein Kinase Tousled-Like 1	
	*UBE3C*	Ubiquitin Protein Ligase E3C	
piR-hsa-23655	*CHURC1*	Churchill Domain Containing 1	
piR-hsa-28646	*LINC01015*	Long Intergenic Non-Protein Coding RNA 1015	
	*ZNF668*	Zinc Finger Protein 668	

## Supplementary Materials

The following are available online at https://www.mdpi.com/article/10.3390/cells11233833/s1, Table S1: miRNA analysis iPSCs vs. Fibroblasts, Table S2: piRNA analysis iPSCs vs. Fibroblasts. Table S3: miRNA analysis Fibroblast high reprogramming efficiency vs. normal reprogramming efficiency. Table S4: mRNA analysis Fibroblast high reprogramming efficiency vs. normal reprogramming efficiency. Table S5: miRNA analysis iPSCs high reprogramming efficiency vs. normal reprogramming efficiency. Table S6: mRNA analysis iPSCs high reprogramming efficiency vs. normal reprogramming efficiency. Table S7: miRNA target filter in fibroblasts with high reprogramming efficiency vs. normal reprogramming efficiency. Table S8: miRNA target filter in iPSCs with high reprogramming efficiency vs. normal reprogramming efficiency.
